# Detection of nanoscale electron spin resonance spectra demonstrated using nitrogen-vacancy centre probes in diamond

**DOI:** 10.1038/ncomms10211

**Published:** 2016-01-05

**Authors:** L. T. Hall, P. Kehayias, D. A. Simpson, A. Jarmola, A. Stacey, D. Budker, L. C. L. Hollenberg

**Affiliations:** 1 School of Physics, University of Melbourne, Parkville, Victoria 3010, Australia; 2 Department of Physics, University of California, Berkeley, California 94720, USA; 3 Centre for Quantum Computation and Communication Technology, School of Physics, University of Melbourne, Parkville, Victoria 3010, Australia

## Abstract

Electron spin resonance (ESR) describes a suite of techniques for characterizing electronic systems with applications in physics, chemistry, and biology. However, the requirement for large electron spin ensembles in conventional ESR techniques limits their spatial resolution. Here we present a method for measuring ESR spectra of nanoscale electronic environments by measuring the longitudinal relaxation time of a single-spin probe as it is systematically tuned into resonance with the target electronic system. As a proof of concept, we extracted the spectral distribution for the P1 electronic spin bath in diamond by using an ensemble of nitrogen-vacancy centres, and demonstrated excellent agreement with theoretical expectations. As the response of each nitrogen-vacancy spin in this experiment is dominated by a single P1 spin at a mean distance of 2.7 nm, the application of this technique to the single nitrogen-vacancy case will enable nanoscale ESR spectroscopy of atomic and molecular spin systems.

## Abstract (web summary)

The application of resonant electron spin ensembles to the study electronic systems can be limited in spatial resolution above the micrometre scale. Here, the authors use nitrogen vacancies to probe the paramagnetic background in diamond via spin resonance spectra dominated by single local spins.

## Graphical abstract

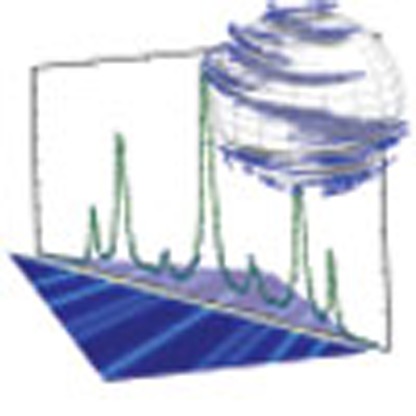

Techniques to detect electron spin resonance (ESR) have long been used to study materials and systems containing unpaired electron spins, such as metal complexes and organic radicals. From an operational viewpoint, the low spin density and high decay rates of such radicals *in-situ*, together with the limited sensitivity of ESR detection systems, often makes the task of obtaining an ESR signal above the detection limit difficult. At present, the best spatial resolution achieved using conventional induction-based ESR detection of solid samples is at the micron level^1^. This is further complicated by the fact that a major component of any biological tissue is water, which has an absorption band in the same region of the electromagnetic spectrum (microwave) as the signals emitted in ESR experiments^2^. As such, there is a great need for a highly sensitive, highly localized technique, which may be used to obtain ESR spectra from paramagnetic systems without the need for microwave control of the sample.

The nitrogen-vacancy (NV) centre point defect in diamond (see refs 3, 4 for extensive reviews), comprised of a substitutional atomic nitrogen impurity and an adjacent vacancy (Fig. 1a), is a promising candidate for such an ideal nanoscale-ESR probe, and is the focus of this work; although we note that the technique outlined in this work may potentially be realized by using other solid-state spin systems such as phosphorous donors in silicon, or gallium-arsenide and indium-arsenide quantum dots. The energy level scheme of the C_3v_-symmetric NV system (Fig. 1b) consists of ground (^3^A_2_), excited (^3^E) and singlet electronic states. The ground-state spin-1 manifold has three spin sublevels (|0〉, |±1〉), which at zero-field are split by 2.87 GHz. Since these sublevels have different fluorescence intensities upon illumination, we can achieve spin-state readout optically^5^,^6^. The degeneracy between the |±1〉 states may be lifted with the application of an external magnetic field, *B*_0_, with a corresponding separation of 2*γB*_0_ (where *γ*=17.6 × 10^6^ rad s^−1^ G^−1^ or 2.80 MHz G^−1^ is the NV gyromagnetic ratio), permitting all three states to be accessible via microwave control. By isolating either the |0〉↔|+1〉 or |0〉↔|−1〉 transitions, the NV spin system constitutes a controllable, addressable spin qubit.

Much attention has been focused on using measurements of the NV quantum phase interference between its spin sublevels^7^,^8^,^9^,^10^ or dephasing rates^11^,^12^,^13^,^14^,^15^,^16^ to characterize dynamic processes occurring in external magnetic environments. These protocols have been shown to have remarkable sensitivity to frequencies in the kHz–MHz range, and are thus well suited to characterizing nuclear spin environments^17^,^18^,^19^,^20^,^21^,^22^,^23^,^24^. However, to achieve the desired sensitivity to the more rapidly fluctuating (GHz) fields associated with electron spin environments, and more importantly, the ability to be frequency-selective, complex and technologically challenging pulse sequences would be necessary^13^,^25^,^26^.

Alternatively, if a transition frequency of an environmental spin approaches that of the NV spin, they will exchange magnetization and their longitudinal spin relaxation rates will increase. This enables the detection of environmental spins by monitoring changes in NV *T*_1_ times^27^,^28^,^29^,^30^,^31^,^32^. This approach can be more sensitive because NV *T*_1_ times can be up to three orders of magnitude longer than spin-echo-based *T*_2_ times. For example, Steinert, *et al.*^27^ (*T*_1_=1.2 ms, *T*_2_=1.9 μs) demonstrated that relaxation-based sensing gave an improvement in the signal-to-noise ratio (SNR) by a factor of 388 over spin-echo methods for the detection of external Gd^3+^ electron spins.

The large NV zero-field splitting is fortuitous in the present context in that transitions in the ground-state spin triplet manifold are far off-resonance from the ∼MHz transition frequencies of the chief magnetic defects in diamond (nitrogen electron-donor defects, referred to hereafter as P1 centres, and ^13^C nuclei), leaving NV transitions unable to be excited unless brought into resonance using an axial magnetic field (*B*_0_≈512 G, for an electron spin environment). The weak spin–orbit coupling to crystal phonons (and low phonon occupancy, owing to the large Debye temperature of diamond) leads to longitudinal relaxation of the spin state on timescales of roughly *T*_1_∼1–10 ms at room temperature, and hundreds of seconds when limited by NV–NV cross-relaxation at temperatures below ∼20 K (ref. 33). On the other hand, the transverse relaxation time (*T*_2_) of the NV spin is the result of dipole–dipole coupling to other spin impurities in the diamond crystal, which occur on timescales of 0.1–1 μs in type 1b (1 of 4) diamond. Sensing based on the much longer longitudinal spin relaxation times can thus be significantly more sensitive than those based on *T*_2_. Projection-noise statistics dictate that the minimum magnetic field, *b*_min_, detectable over a total interrogation time *T*=*Nτ*, comprised of *N* cycles of dark time *τ*, is proportional to 
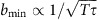
. As the dark time, *τ*, is limited by *T*_1_ in relaxation-based protocols, the latter offer an improvement in sensitivity by a factor of 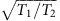 over the former for a fixed interrogation time, *T*. Hence, the relatively long relaxation time of the NV ground state and inherent sensitivity to GHz frequencies, together with room temperature operation and optical readout, make it an ideal system for the ESR spectral mapping protocol discussed in this work.

Here we focus on a new, and potentially simpler, technique for extracting ESR spectra by measuring the longitudinal spin relaxation time, *T*_1_, of a nearby NV centre. Using a controlled external magnetic field, *B*_0_, the transition energies of the NV probe, *ω*_NV_=2*πD*±*γB*_0_, can be brought into resonance with environmental spin energies, *ω*_E_, via the Zeeman effect, and the corresponding dependence of *T*_1_ on *B*_0_ may be measured. We establish a general and robust method to determine the environmental spectral distribution *S*(*ω*_E_) by measuring the relaxation time of the NV probe as a function of the external field strength, *T*_1_(*B*_0_), and discuss how this may be used to perform nanoscale ESR spectroscopy on arbitrary electron spin environments. Our method is demonstrated experimentally by using an ensemble of NV spins to measure the ESR spectrum of the substitutional nitrogen (P1 centres) donor electron spins in type 1b (2 of 4) diamond over the range ±150 MHz. Comparison with theoretical expectations for this known system confirms the validity of this general approach for measuring the ESR spectrum using the NV centre spin probe in a range of nanoscale applications.

## Results

### Relaxation of an NV spin in an arbitrary magnetic environment

**Figure 1:**
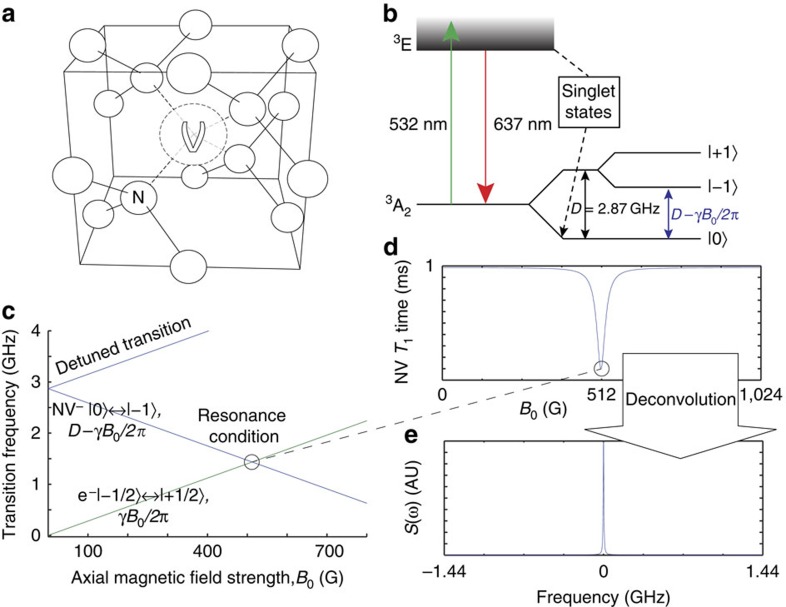
Overview of nitrogen-vacancy and environmental spin energies. (**a**) The nitrogen-vacancy centre point defect in a diamond lattice, comprised of a substitutional nitrogen atom (N) and an adjacent crystallographic vacancy (V). Blank spheres represent carbon atoms. (**b**) The NV ground-state spin sublevels are separated by *D*=2.87 GHz. Upon optical excitation at 532 nm, the population of the |0〉 state may be read out by monitoring the intensity of the emitted red light. (**c**) Transition frequencies of the NV spin, and that of a single electron as a function of external magnetic field strength, *B*_0_. At *B*_0_=512 G, the |0〉↔|−1〉 transition of the NV is resonant with the electron transition, allowing them to exchange energy. (**d**) Measurements of the NV *T*_1_ time versus axial magnetic field strength, *B*_0_, show a dramatic decrease near the *B*_0_=512 G resonance point. (**e**) The inherent broadening of the *T*_1_ resonance in **d** due to the NV–P1 interaction may be removed by deconvolving it from the NV filter function to give the environmental spectral density.

**Figure 2:**
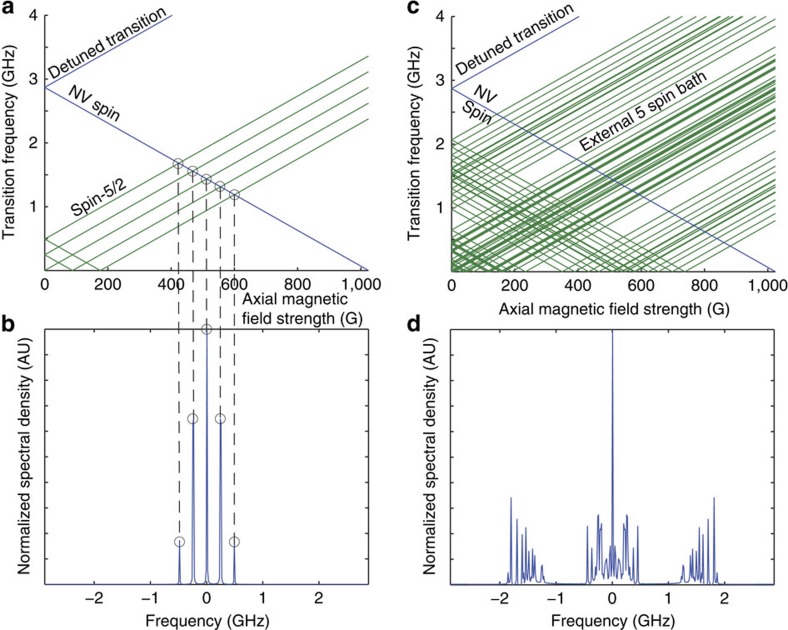
Numerical examples of *T*_1_-based ESR spectroscopy of practically relevant nanoscale environments. (**a**) Transition frequencies of the NV spin and an external spin−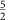 system such as Fe(III) or Mn(II). (**b**) Resulting ESR spectrum of the system in **a**. (**c**) Transition frequencies of the NV spin and an example external system of five randomly placed interacting electrons in a 1 × 1 × 1 nm^3^ cube. (**d**) As in **b** but for the system of five electrons outlined in **c**.

At zero-field, both NV transitions, |0〉↔|+1〉 and |0〉↔|−1〉, occur at ∼2.87 GHz. These transitions are then split symmetrically by the presence of an axial magnetic field and any axial NV–environment couplings, and move to higher and lower frequencies, respectively, with the resulting shift being directly proportional to the strength of the field (Fig. 1b). When the ^3^A_2_ transition frequencies of the spin-1 NV system are brought into resonance with those of a particular environmental frequency (Fig. 1c), the two will be able to exchange polarization via their magnetic dipole interaction. As the axial shifts induced by the environment on the NV spin (typically of order MHz) are constantly fluctuating, they act to broaden the NV's transition frequencies, as characterized by their resulting inhomogeneous linewidth, or transverse spin relaxation rate, Γ_NV_ (which may be either 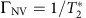 or 1/*T*_2_ depending on the whether or not additional microwave *π* pulses are used to refocus the environment). This results in a resonant enhancement of the NV spin relaxation rates to environmental fields fluctuating within Γ_NV_ of the NV frequency (Fig. 1d), from which the spectral distribution of the environment may be extracted (Fig. 1e). A theoretical example of the case of an NV spin coupled to a spin−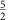 system (such as Mn(II) or Fe(III)) is shown in Fig. 2a,b; and that of a random distribution of five electrons in a 1 × 1 × 1 nm^3^ cube external to the diamond crystal is shown in Fig. 2c,d.

**Figure 3:**
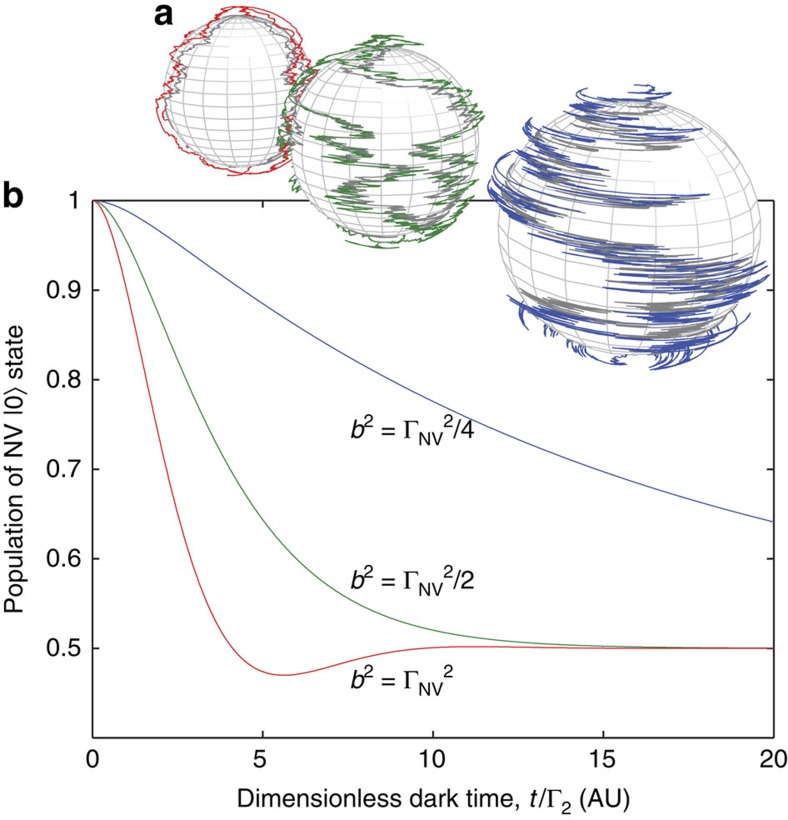
Bloch vector behaviour at constant dephasing rate for three different cases of relative NV–environment couplings. (**a**) Example realizations of Bloch sphere trajectories for cases of under-damped (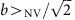, red), critically damped (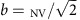, green) and over-damped (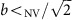, blue) NV–environment couplings, with the +*z* axis representing the |0〉 state of the NV spin, and the −*z* axis representing the |−1〉 state. (**b**) Population of the NV |0〉 state of the NV spin (*P*_0_(*t*)). In the under-damped case the Bloch vector is able to rotate appreciably before its transverse projection decays. In the over-damped case, the transverse projection of the Bloch vector is always pulled back to the *z* axis before it can appreciably rotate. Most practical cases reside in the latter regime because of the intrinsic dephasing arising from strong coupling of the NV to paramagnetic defects in the diamond crystal.

To analytically model the response of the NV spin to such an environment, we must account for two dominant processes: energy exchange between the NV and the environment (effective coupling rate *b*), which changes the population of the magnetic sublevels of the NV ground state; and the destruction of the phase coherence between these sublevels (dephasing, occurring at a rate of Γ_NV_). Depending on the relative strengths of these processes (that is, how many energy exchanges may occur before the NV spin is dephased), the NV spin can exhibit diverse behaviour (Fig. 3). However, as there are typically more sources of dephasing than energy exchange (with the latter effect further decreasing when the NV spin and environment are away from resonance), we have 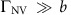 for the cases considered in this work.

Under this regime, if the NV is initially polarized in its |0〉 state and only the |0〉↔|−1〉 transition is being excited, the subsequent population at time *t* when coupled to a spin system of transition energy *ω*_E_ is given by (see Supplementary Note 1 for details)


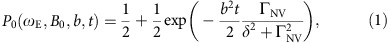


where *δ*=*ω*_NV_−*ω*_E_ is the difference in transition frequencies between the NV (*ω*_NV_) and the environment (*ω*_E_). We note also that this expression does not include the effect of diamond lattice phonons; however, these are included in the analysis of the experimental demonstration below.

Typical electron spin environments will exhibit a distribution of coupling strengths (*b*) and frequencies (*ω*_E_), denoted *P*_*b*_(*b*) and *S*(*ω*_E_, *B*_0_) of roughly the MHz–GHz regime^15^,^28^,^34^, which must be taken into account to analytically determine the full response of an NV ensemble. This response is given by


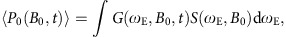


where we identify the function


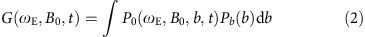


as an environmental spectral filter with a Lorentzian point-spread function centred on the NV transition frequency (equation 1). The NV-relaxation filter, *G*, is tunable via the Zeeman interaction, meaning that it may be directly tuned to specific parts of the environment's spectral distribution, *S*(*ω*_E_, *B*_0_), by choosing the strength of the external magnetic field, *B*_0_.

In the following, we discuss how we may take advantage of the relaxation filter to reconstruct the spectral density, *S*(*ω*_E_, *B*_0_) of an arbitrary environment. If the environment is comprised of some distribution of single electrons, their transition frequencies, *ω*_E_ (including spectral features such as hyperfine interactions, but centred about the *γB*_0_ Zeeman shift), may be brought into resonance with those of the |0〉↔|−1〉 transition of the NV (that is, *ω*_−_∼2*πD−γB*_0_) by choosing a magnetic field strength *B*_0_ such that *B*_0_=*πD*/*γ*≈512 G. In other cases, the environment may contain spin-1 or greater systems possessing their own zero-field splitting (see Fig. 2 for the case of a spin−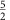 system or (ref. 28) for the case of spin−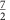 Gd spins coupled to individual NV centres). If the environmental zero-field splittings are greater than that of the NV, the |0〉↔|+1〉 transition of the NV will need to be utilized to ensure that the respective energies are brought into resonance.

### Reconstruction of the environmental spectral density

**Figure 4:**
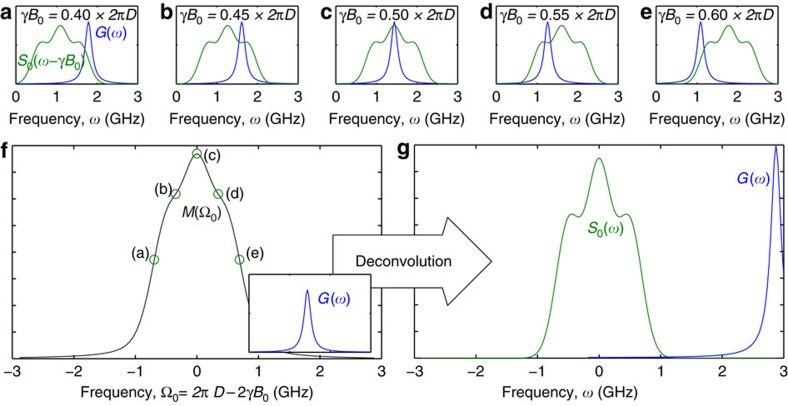
Relationship between the NV filter function, environmental spectral density and the resulting NV-relaxation rate. (**a**–**e**) By controlling the external field strength, *B*_0_, the NV filter function, *G* may be tuned to filter specific regions of the spectral density, *S*. The overlap integral of *G* and *S* is proportional to the relaxation rate at that field strength. (**f**) The resulting measured relaxation rate, *M*(Ω_0_), is the convolution of the NV filter function, *G*, and the spectral density of the environment, *S*. (**g**) Given that *G* is known, the spectral density, *S*, may be reconstructed by deconvolving *S* and *G* from *M*.

As noted above, the region of the spectral density sampled by the NV filter functions may be tuned by controlling the strength of the static external field. This suggests that, by sweeping the filter function across the entire spectrum, we can reconstruct it by measuring the relaxation rate of the NV spin for an appropriate range of external field strengths (see Fig. 4a–e).

We denote an arbitrary given spectrum at zero-field by *S*_0_(*ω*_E_)≡*S*(*ω*_E_, 0). The distribution at some finite external field *B*_0_ is then *S*_0_(*ω*_E_−*γB*_0_). For most cases of practical interest, we assume the shape of the distribution does not change with *γB*_0_, although this case can be handled by extension. Furthermore, we also assume that one of the NV transitions is sufficiently off-resonance that it is not sensitive to the environment, making the overlap with the spectrum insignificant. Even if this is not true, the non-resonant filter function will translate with *γB*_0_ at the same rate as *S*_0_(*ω*_E_−*γB*_0_) (Fig. 1d), and thus produce a constant shift in the overall measurement that does not change with *B*_0_, which may be later subtracted.

The measured response of the NV-relaxation rate, *M*(*ω*_0_, *t*), to *S*_0_ for some external field strength, *B*_0_ (*ω*_0_ ≡ *γB*_0_), is then given by (see Fig. 4f)


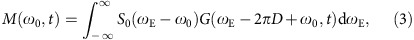


where, for brevity, we have put 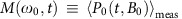. By introducing the frequency-space variable Ω=*ω*_E_−*ω*_0_, and the parameter, Ω_0_=2*πD*−2*ω*_0_, and making use of the symmetry properties of the Lorentzian function, we may write this integral as a Fourier-space convolution,


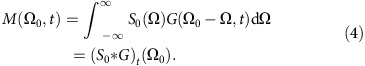


Given that the filter function is known, the spectral density may thus be reconstructed using an appropriate deconvolution algorithm (see Fig. 4g). Owing to the inherent noise that exists within the acquired data set, the Wiener deconvolution method^35^ was found to be ideal for our purposes. An alternative to this would be to fit the data set with an assumed (smooth) functional form, although the accuracy of this approach will depend on having some *a priori* knowledge of the environmental spectrum.

To perform this deconvolution, it is necessary for the function *G*(*ω*_E_) to be known accurately. As this function is the frequency-dependent response of the NV spin to a lateral oscillating magnetic field, *G*(*ω*_E_) may be mapped to arbitrary precision by measuring the response of the NV longitudinal relaxation rate to an applied oscillatory magnetic field of frequency *ω*_E_, provided the microwave field strength, *ω*_*x*_, is such that 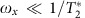 (to avoid microwave broadening of *G*).

In the following section, we provide an experimental example of this protocol applied to the P1 electron spin bath of type 1b (3 of 4) diamond.

### Experimental demonstration

To demonstrate the reconstruction of an environmental spectrum using this technique, we measured the relaxation rate of an ensemble of NV centres subject to a bath of substitutional nitrogen donor (or P1 centre) electron spins. The sample chosen was a synthetic high-pressure high-temperature (HPHT) grown diamond, with ∼50 p.p.m. substitutional nitrogen and 1–10 p.p.m. NV^−^ concentrations.

An NV spin ensemble, as opposed to a single-NV spin, was chosen for the *T*_1_ probe to obtain the average nanoscale P1 spectrum, thereby affording a much more straightforward comparison with the average spectrum discussed in the theoretical component of this work. While the motivation for developing this technique is to use a single-NV spin to perform ESR spectroscopy on proximate nanoscale environments, realization of the P1 distribution surrounding a single-NV centre would exhibit too much variation (owing to the randomized positions occupied by P1 centres relative to a given NV, as well as the different possible orientations and switching timescales of the P1 axes) to perform such a comparison. Despite this, we emphasize that the response of each individual NV centre is the result of its individual nanoscale environment, and that the ensemble-averaged spectrum we obtain is, therefore, the average P1 ESR spectrum due to a nanoscale region surrounding an NV centre.

**Figure 5:**
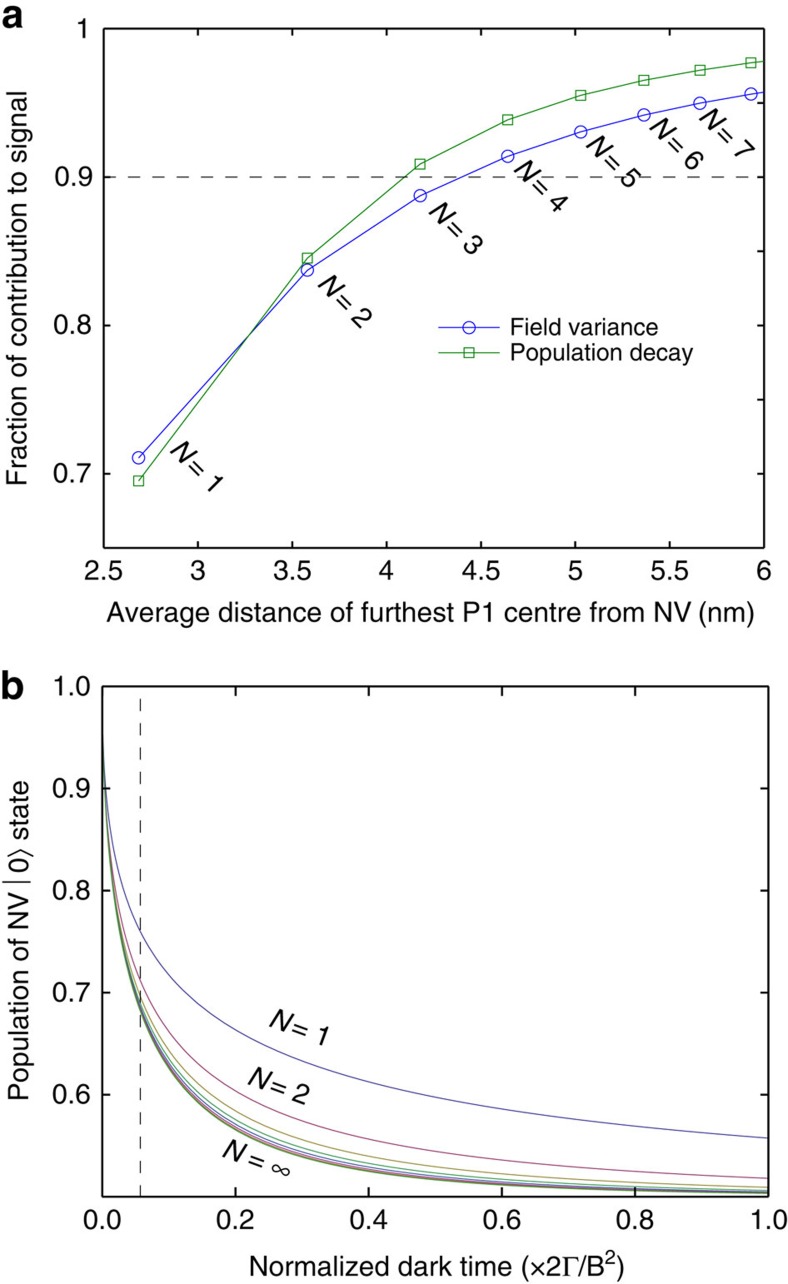
Localization of the effective environmental magnetic field causing relaxation of the NV spin. (**a**) Plot showing the ensemble-averaged fraction of environmental coupling (blue), and decay of the NV |0〉 state population (green), accounted for by considering increasing numbers (*N*) of P1 centres surrounding the NV centre. The horizontal axis shows the averaged distance to the furthest (*N*th) P1 spin. (**b**) Plots showing the ensemble-averaged decay of the NV |0〉 state population due to increasing numbers of P1 centres surrounding the NV centre. The relaxation time of *T*_1_≈0.1 × Γ/*b*^2^ is shown by the dashed line, along which the data for the green curve in (**a**) is obtained.

To estimate the size of this region, we note that spins within it are coupled to the NV spin via the variance of their effective magnetic field strength, *b*^2^ (see equation 1), which is the sum of field variances due to each of the individual P1 spins, 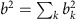. If we order these spins according to their distance from a given NV centre, we may obtain the ensemble-averaged signal by averaging over the probability distributions associated with each successive NV–P1 separation (see Methods section). By examining the average relative contribution of the first to the *N*th P1 spin to the total coupling, 
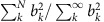
 (Fig. 5a, blue curve), we see that the coupling is dominated by the nearest P1 centre, which resides at an average distance of 2.7 nm from the NV centre, and that 91% of the signal comes from the first four P1 spins surrounding the NV centre with the fourth spin residing at an average distance of 4.6 nm from the NV centre.

The localization of the NV–P1 interaction is seen to be even more pronounced if we consider the entire time-dependent population of the NV |0〉 state rather than the effective field strength alone. This measure is more accurate, since the measured changes in the NV fluorescence are proportional to the population of this state. By decomposing the field variance in the same manner as above, we can see that the exponential term in equation 1 factorizes into individual contributions from each P1 centre. Applying the same ensemble averaging approach as above, we can determine the cumulative contribution to the overall decay of the NV |0〉 state population as each successive P1 centre is incorporated, as shown in Fig. 5b. Finally, by examining this contribution at the relaxation (1/*e*) time of the NV spin, *T*_1_≈0.1 × Γ/*b*^2^, we can see that 91% of the signal is attributable to the nearest three P1 spins, with the third spin residing an average distance of 4.2 nm from the NV centre (Fig. 5a, green curve). These results demonstrate the nanoscale nature of this detection technique. A discussion of the spatial resolution of this technique when applied to ESR spectroscopy of spins external to the diamond crystal may be found in the Discussion section below.

NV centres are oriented along all four symmetry axes, and a solenoid was used to apply an adjustable magnetic field along one of these orientations. After initializing NV spins in the |0〉 state via optical pumping (or the |−1〉 state with an additional microwave *π* pulse), we measured the fluorescence following a variable amount of ‘dark time' (for which the pump laser was off). NVs not aligned with the external field and other defects contribute to the diamond fluorescence, which may decay at rates different from that of the aligned NVs. To remove this effect, we employed a form of common-mode rejection (described in ref. 33). After initializing to either the |0〉 or |−1〉 state, we measured the fluorescence contrast after each dark time, and subtracted the results for each initial state. The resulting decay of the fluorescence difference indicates the loss of NV population from the initial state, and the regions of the environmental spectrum these populations are sensitive to. We note here that for cases in which the application of microwaves may be impractical, this technique may be performed in an all-optical manner, although this approach will also require the characterization of other sources of fluorescence within the sample.

**Figure 6:**
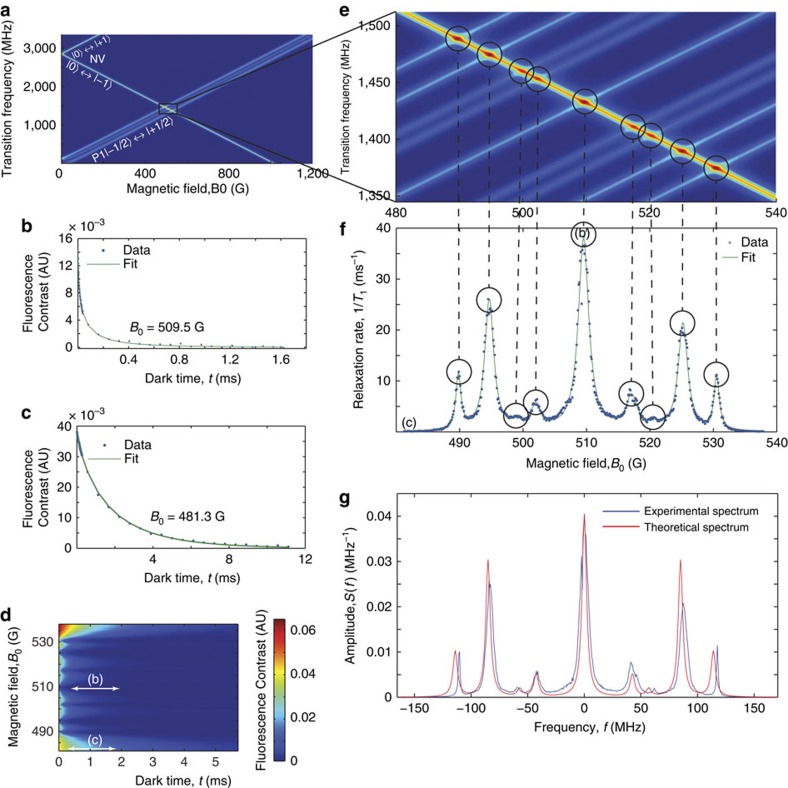
'Experimental demonstration of the reconstruction of an environmental spectrum using an ensemble of NV spins. (**a**) Theoretical plot showing the transition frequencies of the NV centre and P1 electron spins versus the strength of an external field aligned along the 〈111〉 axis. Energy exchange between an NV spin and a nearby P1 spin is achieved when two transition frequencies approach resonance. Broadening of these lines is caused by interactions with other P1 spins and spin impurities within the diamond crystal. (**b**,**c**) Plots of NV centre fluorescence contrast versus dark time at 509.5 and 481.3 G, respectively. In the case of the former, NV and P1 electron energies are on resonance, resulting in a comparatively rapid decay. In the case of the latter, NV and P1 transition energies are too far from resonance to facilitate a dipole-mediated resonant energy exchange, meaning that the depolarization of the NV spin ensemble is dominated by interactions with crystal phonons. (**d**) Measurements of curves such as those shown in **b**,**c** for 500 magnetic field strengths between 480 and 540 G. Data contours used for plots **b**,**c** are highlighted. (**e**) Zoomed plot of that in **a** highlighting the hyperfine structure and the corresponding spin conserving and non-spin conserving transitions of the P1 centre. (**f**) Plot of the external field-dependent relaxation rate, Γ_1_(*B*_0_), extracted from the data shown in **e**. Correspondences of the observed features with the P1 spin transitions in **e** are indicated explicitly by dashed lines, and relaxation rates corresponding to examples in **b**,**c** are labelled as such. (**g**) Application of the deconvolution procedure to the data in **f** yields the spectral density of the spin bath environment surrounding the NV centres in the diamond sample (blue), showing good agreement with theoretical predictions (red).

The P1 centre, the target of our demonstration, is a substitutional defect of a nitrogen atom in place of a carbon atom in a diamond lattice. The single unbonded electron can reside along any one of the four crystallographic bond axes, giving rise to four possible orientations of the hyperfine coupling to the nitrogen nucleus. For field strengths above ∼100 G, the quantization axis of the P1 electron spin is set by its Zeeman interaction, effectively reducing the number of possible P1 species from four to two. Hence, there exists a 25% chance that the delocalization axis of the P1 centre is aligned with the NV axis, giving an axial hyperfine coupling of 114 MHz, and a 75% chance that the delocalization axis is 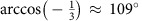 to the NV axis, producing an axial hyperfine coupling of 86 MHz (the spin properties of this defect are discussed in detail in Supplementary Note 2). Figure 6a,b show the overlap of the transition frequencies associated with both the NV spin and the P1 centre.

Measurements of the longitudinal spin relaxation of the NV ensemble, 〈*P*_0_(*t*, *B*_0_)〉_meas_, were taken at 500 different external magnetic field strengths between 480 and 540 G (Fig. 6c–e). Before we deconvolve the data set to determine the spectral density, we extract and inspect the magnetic field-dependent component of the NV-relaxation rate due to spin–spin relaxation with the environment. Accordingly, the data was fitted using the function given by


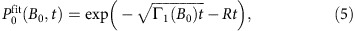


where *R*≈360 Hz is the ‘baseline' component of the relaxation because of diamond lattice phonons (see Methods for the derivation of this fitting form). The resulting spin–spin relaxation rates are plotted in Fig. 6f. As expected, the measurements show that the NV-relaxation rate increases when *D*−2*ω*_0_/2*π*=0, ±86 MHz, ±114 MHz or *B*_0_=490, 495, 510, 525 and 530 G, corresponding to the conditions under which the NV spin may directly exchange magnetization with P1 electron spins, leaving the projection of the P1 nuclear spin unchanged. Other features (as observed previously in (ref. 36)) are evident at *B*_0_=499, 502, 517 and 520 G, and correspond to a two-step process. Firstly, the P1 electron undergoes a mutual flip-flop with the P1 nuclear spin due to their hyperfine interaction. This step is not energy conserving and is heavily suppressed according to the ratio of the hyperfine strength to the strength of the P1 electron's Zeeman interaction. The P1 energy deficit is then paid for by flipping the NV electron spin (as mediated by the *x*−*z* and *y*−*z* components of the NV–P1 electron dipole–dipole interaction—see Supplementary Note 2). Because these transitions are comparatively weak, they are partially obscured by the large spin–phonon relaxation effect of the NV spin, resulting from two-phonon Orbach^37^ and two-phonon^38^ Raman processes.

To obtain the spectral distribution, we carry out the deconvolution over the entire set of data, 〈*P*_0_(*t*, *B*_0_)〉_meas_, and plot the spectral distribution in frequency space, *S*(*f*), in Fig. 6g, demonstrating good agreement with the corresponding theoretical expectations (see Supplementary Note 2 for details). The deconvolution of the spectral density is shown to remove much of the broadening seen in the raw relaxation data in Fig. 6f, and thus provides a better measure of the environmental dynamics. Some small discrepancies between the resulting spectrum and the theoretical result are evident; however, we note that while the theory incorporates effects such as hyperfine couplings and dephasing rates, more complicated effects, such as *g*-factor anisotropy and enhancement, interactions with other paramagnetic impurities and other strain-related phenomena, have not been included. Such effects are highly sample-dependent and thus difficult to predict in general terms, although the technique developed in this work provides an ideal means to facilitate their investigation. Finally, we note that although this demonstration involves ensembles of NV and P1 centres, the detection is highly local as the response of each NV is dominated by its nearest P1 centre: for this sample (50 p.p.m. P1) the mean distance to the nearest P1 centre is about 2.7 nm (ref. 34).

## Discussion

In this work, we have presented a general method for extracting the spectral distribution of an arbitrary electronic environment based on tuning a spin-1 NV probe system, via controlled application of an external field, into resonance with the transitions of the target electronic system. The method was tested using an ensemble of NV centres in a type 1b diamond sample to determine the spectral distribution of the P1 spin bath, showing excellent agreement with the theoretical expectations.

The P1 electron spin bath was chosen as our example environment because it is well understood both theoretically and experimentally, and thus provides an ideal system with which to compare our results; however, practical application of this technique is ultimately intended for spectroscopy of spin systems external to the diamond surface. We note that the ability to couple to such systems is ultimately limited by the intrinsic *T*_1_ of the NV spin: a few milliseconds when phonon limited at room temperature and up to hundreds of seconds when determined by NV–NV interactions at temperatures below 77 K^33^. Such relaxation times would permit room temperature resonance-based spectroscopy of single electron spins up to 100 nm from the NV centre, with distances as far as a few μm possible below 77 K. As NV centres having *T*_1_ times of 1.4 ms have been observed within 3–4 nm of the diamond surface^39^ (with one centre residing stably at 1.9 nm below the surface, although its *T*_1_ time was not reported).

Our relaxation-based ESR method has a number of advantages over the existing techniques. Measurements of the NV relaxation in general do not require microwave control, and thus require no manipulation of the sample. With relaxation times much longer than dephasing times, *T*_1_-based protocols can be significantly more sensitive to ESR detection. Finally, even in the ensemble case demonstrated here, the NV spin relaxation is dominated by local interactions with the environment affords an effective spatial resolution of a few nanometres. By extending this technique to the single-NV probe case as well as other solid-state single-spin systems such as phosphorous donors in silicon and gallium-arsenide quantum dots, determination and characterization of nanoscale ESR spectra of single electronic systems will be possible.

## Methods

### Spatial resolution

The total magnetic variance felt by the NV centre spin is the sum of variances from individual sources,


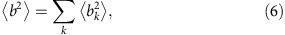


where 
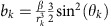
 is the coupling rate to the components of environmental spin *k* that induce NV spin transitions (see Supplementary Note 2 for a derivation of how this term arises from the magnetic dipole–dipole coupling between the NV spin and spins in the environment), *r*_*k*_ is their spatial separation and 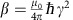.

The probability distribution for the distance from the NV centre to the *k*th nearest P1 centre, *r*_*k*_, is given by^34^


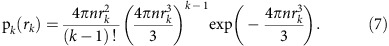


Using this distribution, we find the average distance from a given NV centre to its *k*th nearest P1 centre is given by


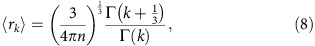


where Γ is the Gamma function, which, for a 50 p.p.m. P1 sample (*n*=8.8 × 10^24^ m^−3^), gives an average distance to the nearest P1 of


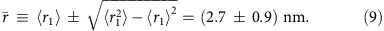


If we substitute equation 6 into equation 1, we see that the time-dependent population of the NV |0〉 state factorizes into individual contributions from each individual field source, *k*. By again ordering these sources in terms of their separation from the NV centre, and averaging over the respective distributions of these separations, we obtain the ensemble-averaged NV |0〉 state population due to field sources 1–*N*,


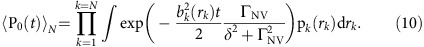


The relative contribution of sources 1–*N* to the overall population decay is then given by 〈P_0_(*t*)〉_*N*_/〈P_0_(*t*)〉_∞_.

### Fitting form

To determine the expected fitting form for the ensemble population of the NV |0〉 state (equation 5), we analytically evaluate the integral in equation 10 for *N*→∞, giving


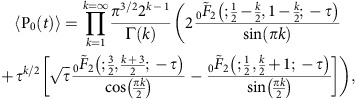


where 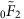 is the regularized, generalized hypergeometric function^40^, and 
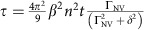
. Expanding about *τ*=0, we find


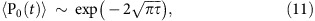


where the dependence of the decay rate on the axial magnetic field, *B*_0_ occurs via the detuning, δ (see main text). To account for the contribution to the NV spin relaxation from environmental phonons, we multiply this result by an additional decay term, exp(−*Rt*), where *R* is independent of the axial field, to give the form used to fit the relaxation data (equation 5).

## Additional information

**How to cite this article:** Hall, L. T. *et al.* Detection of nanoscale electron spin resonance spectra demonstrated using nitrogen-vacancy centre probes in diamond. *Nat. Commun.* 7:10211 doi: 10.1038/ncomms10211 (2016).

## Supplementary Material

Supplementary Information Supplementary Notes 1-2 and Supplementary References.
