# Supplementary material for: Detection of nanoscale electron spin resonance spectra demonstrated using nitrogen-vacancy centre probes in diamond
Source: Nat Commun. 2016 Jan 5;7:10211. doi: 10.1038/ncomms10211 (PMC4728402; doi:10.1038/ncomms10211)
Supplement: Supplementary Information — Supplementary Notes 1-2 and Supplementary References. [file ncomms10211-s1.pdf]

Supplementary Note 1.

Modeling of the NV relaxation process

Here we develop the theory of relaxation based sensing as used in the main text of this work. As we are considering axial magnetic field strengths, B_0, such that $B_0 \sim 2\pi D/2\gamma_e \sim 512\,\text{G}$ (where $D = 2.87\,\text{GHz}$ is the zero-field splitting of the NV spin, and $\gamma_e = 17.6 \times 10^6\,\text{s}^{-1}\,\text{G}^{-1}$ is the electronic gyromagnetic ratio), only the $|0\rangle \leftrightarrow |-1\rangle$ transitions of the NV spin will be appreciably excited, meaning we can disregard any population of the $|+1\rangle$ state. The time evolution of the associated density matrix, ρ_T, is described by

$$\frac{\mathrm{d}\rho_\text{T}}{\mathrm{d}t} = -i\big[\mathcal{H}_\text{T}(t), \rho_\text{T}\big], \tag{1}$$

where ρ_T represents the combined density matrix of the entire NV spin + environment system. The full Hamiltonian is given by $\mathcal{H}_\text{T} = \mathcal{H}_\text{NV} + \mathcal{H}_\text{int} + \mathcal{H}_\text{E}$ where $\mathcal{H}_\text{NV}$ and $\mathcal{H}_\text{E}$ are the self Hamiltonians of the NV centre and environment, which, for a general spin bath environment are given by

$$\begin{aligned} \mathcal{H}_\text{NV} &= 2\pi D\mathcal{P}_z^2 + \omega_\text{NV}\mathcal{P}_z, \\ \mathcal{H}_\text{E} &= \sum_i \left[\omega_\text{E}\mathcal{S}_{z,i} + \sum_{j>i}\mathcal{S}_{z,i}\cdot\mathbf{E}_{ij}\cdot\mathcal{S}_{z,j}\right], \end{aligned} \tag{2}$$

respectively, where we have assumed that the both the z axis and the external magnetic field are aligned with the principle axis of the NV spin, and $\mathbf{E}_{ij}$ is the tensor describing the interaction between spins i and j in the environment, which in general may include both exchange and magnetic-dipole interactions depending on the environment in question.

Due to the highly localised nature of the NV wavefunctions, the coupling of the environmental spins to the NV may be described by the magnetic dipolar interaction alone,

$$\begin{aligned} \mathcal{H}_\text{int} &= \frac{\mu_0}{4\pi}\hbar\gamma_e\gamma_E\sum_i\frac{1}{R_i^3}\left[\vec{\mathcal{P}}\cdot\vec{\mathcal{S}}_i - 3\frac{1}{R_i^2}\left(\vec{\mathcal{P}}\cdot\mathbf{R}_i\right)\left(\mathbf{R}_i\cdot\vec{\mathcal{S}}_i\right)\right], \\ &\equiv \sum_i\left(\vec{\mathcal{P}}\cdot\mathbf{B}_i\cdot\vec{\mathcal{S}}_i\right), \end{aligned} \tag{3}$$

where $\mathbf{B}_i$ is the symmetric magnetic dipole tensor describing the interaction of the NV spin with the ith environmental spin, and includes both transverse and longitudinal components, proportional to $\mathcal{P}_{x,y}$ and $\mathcal{P}_z$ of the NV spin respectively. The latter have a pure dephasing effect, resulting in an additional contribution to the intrinsic dephasing rate of the NV. As relaxation processes occur on timescales that are much longer that the typical interaction timescales of the environmental constituents, essentially placing the system in a Markovian regime, the resulting dephasing will be purely exponential. These effects may thus be modeled using a master equation approach for the reduced density matrix of the NV spin, ρ_NV, as follows

$$\frac{\mathrm{d}\rho_\text{NV}}{\mathrm{d}t} = -i\big[\mathcal{H}(t), \rho_\text{NV}\big] + \left(\mathcal{L}\rho_\text{NV}\mathcal{L}^\dagger - \frac{1}{2}\left\{\mathcal{L}^\dagger\mathcal{L}, \rho_\text{NV}\right\}\right), \tag{4}$$

where, in the present context, $\mathcal{L}$ is the Lindbladian operator corresponding to a pure dephasing process on the NV spin, and is given by $\mathcal{L} = \sqrt{2\Gamma_\text{NV}}\mathcal{P}_z$. The total dephasing rate due to both the local crystal environment and the longitudinal coupling to any external environment is given by $\Gamma_\text{NV} = (T_2^*)^{-1} + \Gamma_2^\text{NV-E}$. The timescale of the intrinsic dephasing process is described using the inhomogeneous linewidth, $(T_2^*)^{-1}$, since the transverse phase accumulation occurs in the absence of any pulsed microwave control. Subtle tuning effects that modify the sensitivity of this technique to various parts of the environmental spectral density may be achieved by changing the intrinsic dephasing rate via dynamic decoupling techniques.

In what follows, owing to the strong intra-environment and comparatively weak NV-environment couplings, we will treat the coupling of the environment to the transverse components of the NV spin as a semiclassical oscillatory field (these simplifications will be later justified in Supplementary Note 2),

$$\begin{aligned} \mathcal{H}_\text{int} &\mapsto \mathcal{B}(\omega_\text{E})\,e^{i\omega_\text{E}t} + \mathcal{B}^\dagger(\omega_\text{E})\,e^{-i\omega_\text{E}t}, \\ &\equiv \mathcal{V} \end{aligned} \tag{5}$$

where $\mathcal{B}\left(\omega_{\mathrm{E}}\right)=b_x\left(\omega_{\mathrm{E}}\right)\mathcal{P}_x+b_y\left(\omega_{\mathrm{E}}\right)\mathcal{P}_y$; and b_x and b_y are the x and y components of the magnetic field. The frequency spectrum (ie the distribution of ω_{E}) is determined by analysing the interaction between environmental constituents, as described by $\mathcal{H}_{\mathrm{E}}$ (again, see Supplementary Note 2). To make the solution tractable, we change to the interaction picture. The transformed equation of motion is given by

$$\frac{\mathrm{d}}{\mathrm{d}t}\rho_I(t) \;=\; -i\big[\mathcal{V}_I(t),\rho_I(t)\big]+\Gamma_{\mathrm{NV}}\big(\mathcal{P}_z\rho_I(t)\mathcal{P}_z-\rho_I(t)\big), \tag{6}$$

with the interaction Hamiltionian given by $\mathcal{V}_I=e^{i\mathcal{H}_{\mathrm{NV}}t}\mathcal{V}e^{-i\mathcal{H}_{\mathrm{NV}}t}$.

We are then interested in determining the rate at which the NV spin relaxes to its equilibrium state under the influence of the environment. We proceed by reducing the 3×3 system of first order linear differential equations described by equation 6 to a higher order differential equation for $P_0\equiv\rho_{00}$. We then wish to solve this equation, together with the initial conditions of $\rho_{ij}=0$ unless $i=j=0$, in which case we have $\rho_{00}=1$, representing the initial polarisation of the NV spin in the $|0\rangle$ state.

To gain insight into the expected analytic solution for the spin-1 NV centre, we consider the simplified case in which only one of the transitions of the NV centre is excited by the environment, and the other is assumed to be too far detuned to have any effect on the population of the spin states. This simplifies the analysis dramatically, yet demonstrates the main properties of relaxation based detection, and is applicable to a spin-1 system for cases of significantly strong Zeeman splittings between the $|\pm1\rangle$ states.

The equation of motion for $P_0(\omega_{\mathrm{NV}},\omega_{\mathrm{E}},b,t)\equiv\rho_{00}(t)$ is

$$\frac{\mathrm{d}^3P_0}{\mathrm{d}t^3}+2\Gamma_{\mathrm{NV}}\frac{\mathrm{d}^2P_0}{\mathrm{d}t^2}+\left(\Gamma_{\mathrm{NV}}^2+\delta^2+\frac{b^2}{2}\right)\frac{\mathrm{d}P_0}{\mathrm{d}t}+\Gamma_{\mathrm{NV}}\frac{b^2}{2}P_0-\Gamma_{\mathrm{NV}}\frac{b^2}{4} \;=\; 0, \tag{7}$$

where $b\equiv\left\langle\mathcal{B}^2\right\rangle^{\frac{1}{2}}$ is the second moment of the strength of the effective magnetic field operator, $\mathcal{B}$, and $\delta=2\pi D-\omega_{\mathrm{NV}}-\omega_{\mathrm{E}}$ is the detuning between the energy levels of the NV ($2\pi D-\omega_{\mathrm{NV}}$) and environmental ($\omega_{\mathrm{E}}$) frequencies.

A. Response to a monochromatic transverse field

1. *Resonant case*

For the case where the frequency of the environment is resonant with the transition frequency between the probe's spin states ($\delta=0$), the solution of Supplementary Equation 7 is

$$P_0(\omega_{\mathrm{NV}},\omega_{\mathrm{E}},b,t) \;=\; \frac{1}{2}+\frac{1}{2}\exp\left(-\frac{\Gamma_{\mathrm{NV}}t}{2}\right)\left[\cosh\left(\frac{t}{4}\sqrt{\Gamma_{\mathrm{NV}}^2-2b^2}\right)+\frac{\Gamma_{\mathrm{NV}}}{\sqrt{\Gamma_{\mathrm{NV}}^2-2b^2}}\sinh\left(\frac{t}{4}\sqrt{\Gamma_{\mathrm{NV}}^2-2b^2}\right)\right]. \tag{8}$$

Typically the spin based environments in which we are interested couple weakly to the NV spin as compared with its intrinsic dephasing rate, implying $\Gamma_{\mathrm{NV}}\gg b$. In fact, even a strong coupling will also induce additional dephasing, so even in a worst case scenario we are guaranteed $\Gamma_{\mathrm{NV}}>b$. In this limit, we have

$$P_0(b,t)|_{\delta=0} \;=\; \frac{1}{2}+\frac{1}{2}\exp\left(-\frac{b^2}{2\Gamma_{\mathrm{NV}}}t\right). \tag{9}$$

Hence the resonant (and therefore maximal) longitudinal relaxation rate is given by

$$\Gamma_1^{\max}=\frac{b^2}{2\Gamma_{\mathrm{NV}}}=\frac{1}{2}T_2^*\left\langle\mathcal{B}^2\right\rangle. \tag{10}$$

2. *General case*

When a finite detuning, δ, exists, we may examine relative importance of the terms within Supplementary Equation 7, subject to rescaling t in terms of the decay time from the resonant solution. That is, if we consider the dimensionless variable $T=\Gamma_1^{\max}t$ and retain terms up to and including order $\mathcal{O}\left(\frac{b^2}{\Gamma_{\mathrm{NV}}^2}\right)$, the solution for an arbitrary detuning becomes

$$P_0(b,t)|_{\delta=0} \;=\; \frac{1}{2}+\frac{1}{2}\exp\left(-\frac{b^2t}{2}\frac{\Gamma_{\mathrm{NV}}}{\Gamma_{\mathrm{NV}}^2+\delta^2}\right). \tag{11}$$

For zero detuning, we recover the previous result (Supplementary Equation 10). For finite detuning, the relaxation rate is modified by a Lorentzian factor with a FWHM of $\Gamma_{\rm NV}$. The complete decay profile is then obtained by integrating this expression over the spectral density of the environment, implying that the δ-dependent relaxation rate acts acts to filter out the environmental spectrum about $\delta \sim 0$.

B. Response to a transverse field with an arbitrary spectral density

Even without considering the specifics of the spectral density, the response of the NV spin to an arbitrary spin bath can vary remarkably due the geometric proximity and arrangement of the bath relative to the NV centre. The definitions of the NV spin relaxation and the corresponding filter function are given by

$$M(\omega_{\rm NV}, t) \;=\; \int G(\omega_{\rm NV}, \omega_{\rm E}, t)\, S(\omega_{\rm NV}, \omega_{\rm E})\, {\rm d}\omega_{\rm E}, \tag{12}$$

,and

$$G(\omega_{\rm NV}, \omega_{\rm E}, t) \;=\; \int P_0(\omega_{\rm NV}, \omega_{\rm E}, b, t) P_b(b)\, {\rm d}b, \tag{13}$$

respectively, where the filter function, $G(\omega_{\rm NV}, \omega_{\rm E})$ acts to filter out regions of the spectral density (as dependent on the external field strength, $B_0 = \omega_{\rm NV}/\gamma_{\rm e}$), and depends explicitly on the geometric arrangement of the environmental constituents. Ultimately, given some measurement record and filter function, it is expression 12 that must be deconvolved to reproduce the spectral density, $S(\omega_{\rm E})$. In this section, we consider the effects of the geometric arrangement of the environment on the filter function, G, for a general spectral density; and specific case of the internal P1 nitrogen donor electron spin bath in type-1b diamond is considered below in Supplementary Note 2.

1. *Response to an internal (bulk) spin bath*

We note that the coupling of the NV to a bath spin located at some distance r may be written $b \equiv \beta(\theta, \phi)/r^3$, where the specific details of the angular dependence of the coupling are incorporated into the parameter $\beta(\theta, \phi)$. We note that is is necessary to omit further discussion of β until Supplementary Note 2, as different environmental processes will be more readily detectable by the NV spin at different relative angles. Unlike the transverse (spin echo) case, where the precession of the NV spin vector in the $x - y$ plane is sensitive to *all* longitudinal field sources, the effect on the longitudinal projection is dominated by the coupling to the nearest P1 electron spin. As such, we may write $P_b(b) = P_r(r) P_{\theta,\phi}(\theta, \phi)$, where r, θ, ϕ are the spherical coordinates associated with the distribution of field sources.

From Ref. 1, the distribution of distances from a given NV centre to its nearest spin impurity is given by

$$\mathrm{P}_r(r) \;=\; 4\pi n r^2 \exp\left(\frac{4}{3}\pi n r^3\right), \tag{14}$$

where n is the average density of impurities in the bath. Substituting this expression into Supplementary Equation 12, we find

$$\begin{aligned} M(\omega_{\rm NV}, t) \;&=\; \frac{1}{2} + \frac{1}{3}\sqrt{2\pi} n \int \beta(\theta,\phi) \sqrt{\frac{\Gamma_{\rm NV} t}{\Gamma_{\rm NV}^2 + \delta^2}}\, \mathfrak{G}^{3,0}_{0,3}\left(\frac{8\beta^2(\theta,\phi) n^2 \pi^2 t \Gamma_{\rm NV}}{9\,(\Gamma_{\rm NV}^2 + \delta^2)} \,\middle|\, -\tfrac{1}{2}, 0, \tfrac{1}{2} \right) \\ &\qquad \times P_{\theta,\phi}(\theta,\phi)\, {\rm d}\theta\, {\rm d}\phi\, S(\omega_{\rm NV}, \omega_{\rm E})\, {\rm d}\omega_{\rm E}, \\ &\sim\; \int \exp\left(-\frac{2\pi}{3} \left(2\pi n^2 t\right)^{1/2} \big\langle |\beta| \big\rangle \sqrt{\frac{\Gamma_{\rm NV}}{\Gamma_{\rm NV}^2 + \delta^2}} \right) S(\omega_{\rm NV}, \omega_{\rm E})\, {\rm d}\omega_{\rm E}, \end{aligned} \tag{15}$$

where $\mathfrak{G}$ is the Meijer-G function, and $\big\langle |\beta| \big\rangle = \int |\beta(\theta,\phi)|\, P_{\theta,\phi}(\theta,\phi)\, {\rm d}\theta\, {\rm d}\phi$. Thus, we identify the filter function associated with environments inside the diamond lattice to be

$$G_{\rm in}(\omega_{\rm NV}, \omega_{\rm E}) \;=\; A_{\rm in} \sqrt{\frac{\Gamma_{\rm NV}}{(2\pi D - \omega_{\rm NV} - \omega_{\rm E})^2 + \Gamma_{\rm NV}^2}}, \tag{16}$$

where $A_{\rm in}$ is a constant, associated with the geometry of the bath, that may be renormalised.

2. Response to an external (surface) spin bath

In contrast to the bulk spin bath case, spins on the surface are unable to exist arbitrarily close to the NV centre. Typically we consider samples in which NV centres exist at some depth $h + \delta h$ below the surface, with h being the mean depth, and δh a normally distributed variable with variance $\left\langle \delta h^2 \right\rangle \ll h^2$. In this case, an individual NV spin is exposed to many bath spins, meaning that the effective coupling distribution, $\mathrm{P}_b(b)$, is normally distributed.

In this case, we may expand Supplementary Equation 11 for small t, which, upon substitution into Supplementary Equation 12 and averaging over $\mathrm{P}_b(b)$ gives

$$\left\langle P_0(t) \right\rangle \;\sim\; 1 - t \left\langle b^2 \right\rangle \left(\int \frac{\Gamma_{\mathrm{NV}}}{\Gamma_{\mathrm{NV}}^2 + \delta^2} \, S(\omega_{\mathrm{NV}}, \omega_{\mathrm{E}}) \, \mathrm{d}\omega_{\mathrm{E}} \right), \tag{17}$$

Thus, we identify the filter function associated with environments outside the diamond lattice to be

$$G_{\mathrm{out}}(\omega_{\mathrm{E}}, \omega_0) \;=\; A_{\mathrm{out}} \frac{\Gamma_{\mathrm{NV}}}{(2\pi D - \omega_{\mathrm{NV}} - \omega_{\mathrm{E}})^2 + \Gamma_{\mathrm{NV}}^2}. \tag{18}$$

Supplementary Note 2.

Theoretical description of the coupled NV-P1 system

In this section, we discuss the features we expect to be evident in the P1 centre spectrum by examining the effect of a P1 centre on the magnetic field dependent relaxation rate of a near-by NV centre. We conclude this section by demonstrating the equivalence of the semi-classical approach used in this work, and a quantum mechanical treatment of the NV-P1 interaction.

A. P1 Hamiltonian

The Hamiltonian of a P1 centre is given by

$$\mathcal{H}_{\mathrm{P1}} = \vec{\mathcal{S}} \cdot \mathbf{A}_{\mathrm{P1}} \cdot \vec{\mathcal{I}} + \mathbf{B}_0 \cdot \left(\gamma_{\mathrm{e}} \vec{\mathcal{S}} + \gamma_{\mathrm{N}} \vec{\mathcal{I}}\right) + \vec{\mathcal{I}} \cdot \mathbf{Q}_{\mathrm{P1}} \cdot \vec{\mathcal{I}}, \tag{19}$$

where $\mathbf{A}_{\mathrm{P1}}$ is the hyperfine tensor describing the coupling between the P1 electron, $\vec{\mathcal{S}}$, and ^{14}N nuclear spin, $\vec{\mathcal{I}}$; and $\mathbf{Q}$ is the quadrupole splitting of the nuclear spin. For all field strengths at which there is an appreciable overlap of this spectrum with the NV spin filter function, $B_0 \sim 512\,\mathrm{G}$, we find that the eigenstates of the P1 centre electron spin are predominantly dictated by the external magnetic field. In this instance, the Hamiltonian of the P1 centre becomes

$$\mathcal{H}_{\mathrm{P1}}^{\mathrm{on}} = A_z \mathcal{S}_z \mathcal{I}_z + A_x \left(\mathcal{S}_x \mathcal{I}_x + \mathcal{S}_y \mathcal{I}_y\right) + B_0 \left(\gamma_{\mathrm{e}} \mathcal{S}_z + \gamma_{\mathrm{N}} \mathcal{I}_z\right) + Q_{\mathrm{P1}} \mathcal{I}_z^2, \tag{20}$$

for cases where the P1 axis is aligned with the external magnetic field, where $A_z = 114\,\mathrm{MHz}$, and $A_x = 81.3\,\mathrm{MHz}$. If the P1 axis is aligned along one of the three other bond directions not aligned with the field, the Hamiltonian may be transformed via the rotation operator $\mathcal{R} = \exp\left(-i\mathcal{I}_y\theta\right) = 1 - i\mathcal{I}_y \sin(\theta) - \mathcal{I}_y^2 \left(1 - \cos(\theta)\right)$ (The other two axes may be realised via a trivial rotation about the z axis), and is given by

$$\mathcal{H}_{\mathrm{P1}}^{\mathrm{off}} = a_z \mathcal{S}_z \mathcal{I}_z + a_x \left(\mathcal{S}_x \mathcal{I}_x + \mathcal{S}_y \mathcal{I}_y\right) + B_0 \left(\gamma_{\mathrm{e}} \mathcal{S}_z + \gamma_{\mathrm{N}} \mathcal{I}_z\right) + Q'_{\mathrm{P1}} \mathcal{I}_z^2, \tag{21}$$

where $a_z = \frac{1}{9}\left(8A_x + A_z\right) = 85\,\mathrm{MHz}$, and $a_x = \frac{1}{9}\left(5A_x + 4A_z\right) = 99\,\mathrm{MHz}$.

B. Coupling of the P1 environment to the NV centre

The interaction between the NV spin and the P1 nuclear spin is ignored on account of its comparative weakness. For the interaction between the NV and the P1 electron, the magnetic dipole interaction is given by

$$\mathcal{H}_{\mathrm{int}} = b_{\mathrm{int}} \left[\vec{\mathcal{P}} \cdot \vec{\mathcal{S}} - 3\left(\hat{\mathbf{r}} \cdot \vec{\mathcal{P}}\right)\left(\hat{\mathbf{r}} \cdot \vec{\mathcal{S}}\right)\right], \tag{22}$$

where $\hat{\mathbf{r}}$ is the unit separation vector between the NV and P1 centres, and

$$b_{\mathrm{int}} = \frac{\mu_0 \hbar \gamma_{\mathrm{e}}^2}{4\pi r^3}, \tag{23}$$

is the effective dipolar coupling strength.

The components of the NV-P1 interaction responsible for the relaxation of the NV spin are those coupling to its transverse components, namely $\mathcal{P}_x$ and $\mathcal{P}_y$. Without loss of generality, we may rewrite this interaction as an effective quantum mechanical magnetic field, $\vec{\mathcal{B}}$, where $\mathcal{B}_{x,y,z}$ couples to $\mathcal{P}_{x,y,z}$.

To make use of Supplementary Equation12, we make the semi-classical approximation, and assume that NV-P1 interaction plays very little part in determining the dynamics of the environment. The problem of determining the environmental spectrum then reduces to solving for the environmental evolution exclusively under its own influence as follows.

In order to determine the effective strength of the semi-classical magnetic field used to model the effect of the P1 bath on the NV spin, we must determine which components of the NV-P1 interaction, $\mathcal{H}_{\mathrm{int}}$, are relevant, and which may be discarded for a given magnetic field strength. To do this, we transform $\mathcal{H}_{\mathrm{int}}$ to the interaction picture (we point out that only the Hilbert space associated with the NV and P1 electron spins need be considered, as the

interaction between the NV electron spin and the P1 nuclear spin is sufficiently weak that it may be ignored). In matrix form, $\mathcal{H}_{\text{int}}$ is given (in the $\left\{\left|0,+\frac{1}{2}\right\rangle,\left|0,-\frac{1}{2}\right\rangle,\left|-1,+\frac{1}{2}\right\rangle,\left|-1,-\frac{1}{2}\right\rangle\right\}$ basis) by

$$\mathcal{H}_{\text{int}} \cong \frac{1}{2\sqrt{2}}\begin{pmatrix} 0 & 0 & b_{xz}-ib_{yz} & b_{xx}-2ib_{xy}-b_{yy} \\ 0 & 0 & b_{xx}+b_{yy} & -b_{xz}+ib_{yz} \\ b_{xz}+ib_{yz} & b_{xx}+b_{yy} & -\sqrt{2}b_{zz} & -\sqrt{2}\left(b_{xz}-ib_{yz}\right) \\ b_{xx}+2ib_{xy}-b_{yy} & -b_{xz}-ib_{yz} & -\sqrt{2}\left(b_{xz}+ib_{yz}\right) & \sqrt{2}b_{zz} \end{pmatrix}. \tag{24}$$

In order to simplify $\mathcal{H}_{\text{int}}$, we switch to the interaction picture to see which terms are important near 512 G.

$$\begin{aligned}\mathcal{H}^{(\text{I})}_{\text{int}} &= e^{i(\mathcal{H}_{\text{NV}}+\mathcal{H}_{\text{P1}})t}\mathcal{H}_{\text{int}}e^{-i(\mathcal{H}_{\text{NV}}+\mathcal{H}_{\text{P1}})t} \\ &\cong \frac{1}{2\sqrt{2}}\left(\begin{matrix} 0 & 0 \\ 0 & 0 \\ e^{it(D-\omega_0)}\left(b_{xz}+ib_{yz}\right) & e^{it(D+kA_z)}\left(b_{xx}+b_{yy}\right) \\ e^{it(D-kA_z-2\omega_0)}\left(b_{xx}+2ib_{xy}-b_{yy}\right) & -e^{it(D-\omega_0)}\left(b_{xz}+ib_{yz}\right) \end{matrix}\right. \cdots \\ &\qquad \cdots \left.\begin{matrix} e^{-it(D-\omega_0)}\left(b_{xz}-ib_{yz}\right) & e^{-it(D-kA_z-2\omega_0)}\left(b_{xx}-2ib_{xy}-b_{yy}\right) \\ e^{-it(D+kA_z)}\left(b_{xx}+b_{yy}\right) & -e^{-it(D-\omega_0)}\left(b_{xz}-ib_{yz}\right) \\ -\sqrt{2}b_{zz} & -\sqrt{2}e^{it(kA_z+\omega_0)}\left(b_{xz}-ib_{yz}\right) \\ -\sqrt{2}e^{-it(kA_z+\omega_0)}\left(b_{xz}+ib_{yz}\right) & \sqrt{2}b_{zz} \end{matrix}\right), \end{aligned} \tag{25}$$

where $k=-1,0,+1$ is the hyperfine projection of the nuclear spin. Using the rotating wave approximation, we can see that only terms of frequency $2\pi D-2\omega_0-2\pi kA_z$ need be retained, and all other off-diagonal terms may be ignored. Transforming back to the Schrodinger picture, the simplified interaction Hamiltonian becomes

$$\mathcal{H}_{\text{int}} \cong \frac{1}{2\sqrt{2}}\begin{pmatrix} 0 & 0 & 0 & b_{xx}-2ib_{xy}-b_{yy} \\ 0 & 0 & 0 & 0 \\ 0 & 0 & -\sqrt{2}b_{zz} & 0 \\ b_{xx}+2ib_{xy}-b_{yy} & 0 & 0 & \sqrt{2}b_{zz} \end{pmatrix}, \tag{26}$$

from which we infer that the effective transverse field strength associated with the allowed NV-P1 $|0,\downarrow\rangle \leftrightarrow |+1,\uparrow\rangle$ transitions ((a), (b) in Supplementary Figure 1) is given by

$$\begin{aligned} b_{\text{all}} &\equiv \frac{1}{2}\sqrt{\left(b_{xx}+b_{yy}\right)^2+\left(2b_{xy}\right)^2} \\ &= \frac{3}{2}b\sin^2(\Theta). \end{aligned} \tag{27}$$

Determination of the effective field strength associated with the disallowed transitions ((c), (d) in Supplementary Figure 1) follows the same approach as above. From this, we find the associated field strength to be

$$\begin{aligned} b_{\text{dis}} &\equiv \frac{1}{2}\sqrt{b_{xz}^2+b_{yz}^2}, \\ &= \frac{3}{4}b\sin(2\Theta). \end{aligned} \tag{28}$$

C. P1 dynamics

To determine the dynamic behaviour of the P1 environment, we compute the autocorrelation functions associated with the field components above. Interactions between environmental components may be modeled by damping these autocorrelation functions with a decaying exponential, $\exp(-\Gamma_{\text{P1}}t)$ to describe their relaxation due to mutual flip-flop processes with corresponding relaxation rate Γ_{P1}.

The corresponding spectra may then be found by computing the Fourier transforms of the autocorrelation functions. From this, we find the spectra associated with the allowed transitions to be

$$S^{\text{on}}_{\text{all}}(\omega) = \frac{1}{6\pi}\left[\frac{\Gamma_{\text{P1}}}{\Gamma_{\text{P1}}^2+(\omega\pm\omega_0)^2}+\frac{\Gamma_{\text{P1}}}{\Gamma_{\text{P1}}^2+(\omega\pm\omega_0+A_z)^2}+\frac{\Gamma_{\text{P1}}}{\Gamma_{\text{P1}}^2+(\omega\pm\omega_0-A_z)^2}\right], \tag{29}$$

and

$$S^{\rm off}_{\rm all}(\omega) \;=\; \frac{1}{6\pi}\left[\frac{\Gamma_{\rm P1}}{\Gamma^2_{\rm P1}+(\omega\pm\omega_0)^2}+\frac{\Gamma_{\rm P1}}{\Gamma^2_{\rm P1}+(\omega\pm\omega_0+a_z)^2}+\frac{\Gamma_{\rm P1}}{\Gamma^2_{\rm P1}+(\omega\pm\omega_0-a_z)^2}\right], \tag{30}$$

for cases of on and off axis P1 centres respectively. Taking the relative proportions of on and off-axis P1 centres to be 25% and 75% respectively, we find the overall spectrum associated with the allowed transitions to be

$$S_{\rm all}(\omega) \;=\; \frac{1}{4}S^{\rm on}_{\rm all}(\omega)+\frac{3}{4}S^{\rm off}_{\rm all}(\omega). \tag{31}$$

Similarly, the spectra associated with the disallowed transitions are given by

$$S^{\rm on}_{\rm dis}(\omega) \;=\; \frac{1}{4\pi}\left[\frac{\Gamma_{\rm P1}}{\Gamma^2_{\rm P1}+(\omega\pm\omega_0+\lambda_1)^2}+\frac{\Gamma_{\rm P1}}{\Gamma^2_{\rm P1}+(\omega\pm\omega_0-\lambda_2)^2}\right], \tag{32}$$

and

$$S^{\rm off}_{\rm dis}(\omega) \;=\; \frac{1}{4\pi}\left[\frac{\Gamma_{\rm P1}}{\Gamma^2_{\rm P1}+(\omega\pm\omega_0+\lambda_3)^2}+\frac{\Gamma_{\rm P1}}{\Gamma^2_{\rm P1}+(\omega\pm\omega_0-\lambda_4)^2}\right], \tag{33}$$

respectively, where

$$\begin{aligned}
\lambda_1 &= \sqrt{2A_x^2+\left(\omega_0+\frac{A_z}{2}\right)^2}\sim\omega_0+\frac{A_z}{2}\\
\lambda_2 &= \sqrt{2A_x^2+\left(\omega_0-\frac{A_z}{2}\right)^2}\sim\omega_0-\frac{A_z}{2}\\
\lambda_3 &= \sqrt{2a_x^2+\left(\omega_0+\frac{a_z}{2}\right)^2}\sim\omega_0+\frac{a_z}{2}\\
\lambda_4 &= \sqrt{2a_x^2+\left(\omega_0-\frac{a_z}{2}\right)^2}\sim\omega_0-\frac{a_z}{2}.
\end{aligned}$$

The spectrum associated with the disallowed transitions is then

$$S_{\rm dis}(\omega) \;=\; \frac{1}{4}S^{\rm on}_{\rm dis}(\omega)+\frac{3}{4}S^{\rm off}_{\rm dis}(\omega). \tag{34}$$

By employing the full spectrum, $S(\omega_E)=S_{\rm all}(\omega_E)+S_{\rm dis}(\omega_E)$, in equation 12, we find the resulting external field-dependent relaxation rate of the NV centre to be

$$\begin{aligned}
\Gamma_1(\omega_0) \;=\; & \frac{\langle b_\perp^2\rangle}{6\pi}\Bigg[\frac{1}{4}\left(\frac{\Gamma_2+\Gamma_{\rm P1}}{(\Gamma_2+\Gamma_{\rm P1})^2+4(\omega_0\pm\frac{D}{2}+\frac{A_z}{2})^2}+\frac{\Gamma_2+\Gamma_{\rm P1}}{(\Gamma_2+\Gamma_{\rm P1})^2+4(\omega_0\pm\frac{D}{2}-\frac{A_z}{2})^2}\right)\\
&+\frac{3}{4}\left(\frac{\Gamma_2+\Gamma_{\rm P1}}{(\Gamma_2+\Gamma_{\rm P1})^2+4(\omega_0\pm\frac{D}{2}+\frac{a_z}{2})^2}+\frac{\Gamma_2+\Gamma_{\rm P1}}{(\Gamma_2+\Gamma_{\rm P1})^2+4(\omega_0\pm\frac{D}{2}-\frac{a_z}{2})^2}\right)\\
&+\frac{\Gamma_2+\Gamma_{\rm P1}}{(\Gamma_2+\Gamma_{\rm P1})^2+4(\omega_0\pm\frac{D}{2})^2}\Bigg]\\
&+\frac{\langle b_\parallel^2\rangle}{4\pi}\Bigg[\frac{1}{4}\left(\frac{\Gamma_2+\Gamma_{\rm P1}}{(\Gamma_2+\Gamma_{\rm P1})^2+4(\omega_0\pm\frac{D}{2}+\frac{A_z}{4})^2}+\frac{\Gamma_2+\Gamma_{\rm P1}}{(\Gamma_2+\Gamma_{\rm P1})^2+4(\omega_0\pm\frac{D}{2}-\frac{A_z}{4})^2}\right)\\
&+\frac{3}{4}\left(\frac{\Gamma_2+\Gamma_{\rm P1}}{(\Gamma_2+\Gamma_{\rm P1})^2+4(\omega_0\pm\frac{D}{2}+\frac{a_z}{4})^2}+\frac{\Gamma_2+\Gamma_{\rm P1}}{(\Gamma_2+\Gamma_{\rm P1})^2+4(\omega_0\pm\frac{D}{2}-\frac{a_z}{4})^2}\right)\Bigg],
\end{aligned} \tag{35}$$

where the effective couplings, $\langle b_\perp^2\rangle$ and $\langle b_\parallel^2\rangle$, are due to integration over all possible NV-P1 separations. By taking the average P1 density to be 50 ppm, and the FID rate to be $\Gamma_2 = 5.0\,$MHz, we may plot the resulting field-dependent relaxation rate of the NV spin (Supplementary Figure 1).

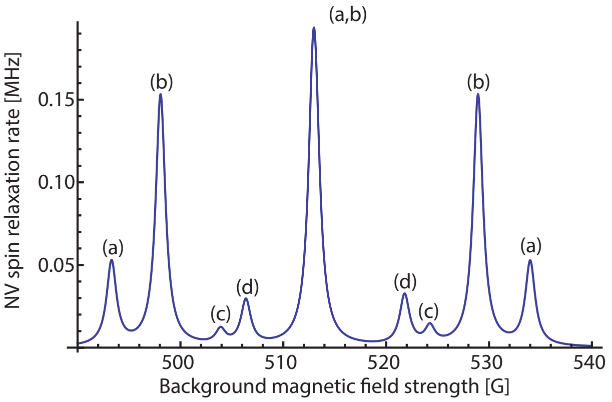

Supplementary Figure 1: Analytic calculation of the relaxation rate of an NV centre spin placed in a 50ppm environment of P1 donor electron spins vs the strength of an external magnetic field aligned along the NV axis. (a) Peaks associated with allowed transitions of the NV centre spin due to its direct interaction with aligned P1 centre electron spins. (b) As in (a), but for the case of P1 centres not aligned along the NV axis. (c) Peaks associated with disallowed transitions of the NV spin as mediated by flip-flop dynamics between the on-axis P1 electron and nuclear spins. (d) As in (c), but for the case of off-axis P1 centres.

D. Quantum mechanical justification of the semi-classical approach

In the analysis above, we have concerned ourselves with the response of the NV spin to a general transverse classical field. Whilst the spectral makeup of this field is entirely general, and thus applicable to the sensing of fields external to the diamond crystal (the primary motivation of this work), it still remains to be shown the that quantum mechanical P1 environment used as our practical example system can also be modelled using this technique. We show this by modelling the NV-P1 system exactly, using the same dephasing model as the semi-classical approach above. As the two approaches produce identical results, we conclude that the semi-classical approach to modelling the P1 bath, and the resulting filter-function picture employed in the analysis of experimental results, is valid.

Given the discussion above about the static spin projection of the P1 nuclear spin projection, we may regard its influence on the P1 electron spin as an additional static magnetic field, where the total field felt by the P1 electron is given by $\omega_{\mathrm{P1}} = \omega_0 + 2\pi k A$, where A is the P1 hyperfine coupling, and $k = -1, 0, +1$. Thus, we may restrict our system to the NV ($\mathcal{P}$) and P1 ($\mathcal{S}$) electron spins.

The Hamiltonian of the system is

$$\mathcal{H}_{\mathrm{NV-P1}} = \mathcal{H}_0 + \mathcal{H}_{\mathrm{int}}, \tag{36}$$

where

$$\mathcal{H}_0 = 2\pi D \mathcal{P}_z^2 + \omega_{\mathrm{NV}} \mathcal{P}_z + \omega_{\mathrm{P1}} \mathcal{S}_z, \tag{37}$$

and $\mathcal{H}_{\mathrm{int}}$ is as previously defined in Supplementary Equation 3.

Following the same procedure as in the semi-classical case, we assume that both the NV and P1 electron spins experience a dephasing effect from the distant components of the bath, with corresponding rates given by Γ_{NV} and Γ_{P1} respectively. The equation of motion for the full (NV and P1) density matrix in the interaction picture is then

$$\frac{\mathrm{d}}{\mathrm{d}t}\rho_I(t) = -i\big[\mathcal{V}_I(t), \rho_I(t)\big] + \Gamma_{\mathrm{NV}}\big(\mathcal{P}_z \rho_I(t) \mathcal{P}_z - \rho_I(t)\big) + \Gamma_{\mathrm{P1}}\big(\mathcal{S}_z \rho_I(t) \mathcal{S}_z - \rho_I(t)\big), \tag{38}$$

with the interaction Hamiltionian given by

$$\mathcal{V}_I = e^{i\mathcal{H}_0 t} \mathcal{V} e^{-i\mathcal{H}_0 t}. \tag{39}$$

There are two basis states associated with the $|0\rangle$ state of the NV, namely $\left|0, +\frac{1}{2}\right\rangle$ and $\left|0, -\frac{1}{2}\right\rangle$, with corresponding populations given by the density matrix elements ρ_{33} and ρ_{44} respectively. The corresponding equations of motion are

$$\frac{\mathrm{d}\rho_{33}}{\mathrm{d}t} = 0,$$

$$\frac{\mathrm{d}^3\rho_{44}}{\mathrm{d}t^3} + 2\left(\Gamma_{\mathrm{NV}} + \Gamma_{\mathrm{P1}}\right)\frac{\mathrm{d}^2\rho_{44}}{\mathrm{d}t^2} + \left[\frac{b^2}{2} + \delta^2 + \left(\Gamma_{\mathrm{NV}} + \Gamma_{\mathrm{P1}}\right)^2\right]\frac{\mathrm{d}\rho_{44}}{\mathrm{d}t} + \frac{b^2}{2}\left(\Gamma_{\mathrm{NV}} + \Gamma_{\mathrm{P1}}\right)\rho_{44} - \frac{b^2}{4}\left(\Gamma_{\mathrm{NV}} + \Gamma_{\mathrm{P1}}\right) = 0, \tag{40}$$

where, as in the semi-classical approximation, we have made the substitution $b \equiv \frac{1}{2}\sqrt{(b_{xx}+b_{yy})^2+(2b_{xy})^2}$.

As can be seen by comparison with Supplementary Equation 7, the semi-classical and quantum mechanical approaches yield an identical differential equation, up to a replacement of $\Gamma_{\rm NV} \mapsto \Gamma_{\rm NV} + \Gamma_{\rm P1}$. Physically, this relates the total broadening of the NV ralaxation rate, to the intrinsic NV linewidth, $\Gamma_{\rm NV}$, and the linewidth of P1 electron spin, $\Gamma_{\rm P1}$, in the same way that the broadening of the NV relaxation rate in the semiclassical picture is the convolution of the NV filter function (having width $\Gamma_{\rm NV}$) and the spectral density of the P1 environment (having width $\Gamma_{\rm P1}$).

Supplementary References

[1] Hall, L. T., Cole, J. H. & Hollenberg, L. C. L. Analytic solutions to the central-spin problem for nitrogen-vacancy centers in diamond. *Physical Review B* **90**, 075201 (2014).
